# Evidence-based guidelines for greener, healthier, more resilient neighbourhoods: Introducing the 3–30–300 rule

**DOI:** 10.1007/s11676-022-01523-z

**Published:** 2022-08-26

**Authors:** Cecil C. Konijnendijk

**Affiliations:** Nature Based Solutions Institute, Barcelona, Spain

**Keywords:** Climate adaptation, Guidelines, Public health, Trees, Urban forestry

## Abstract

The important contributions of urban trees and green spaces to for example, climate moderation and public health have been recognized. This paper discusses guidelines and norms that promote the benefits of viewing green, living amongst green, and having easy access to green spaces for recreational use. Having trees and other vegetation in sight from one’s home, place of work, or school has important mental health and performance benefits. Local tree canopy cover is positively associated with cooling and other aspects of climate moderation. With public green spaces in proximity to one’s home stimulates regular use of these areas and results in positive impacts on mental, physical, and social health. After analyzing existing guidelines and rules for urban green space planning and provision, a new, comprehensive guideline is presented, known as the ‘3–30–300 rule’ for urban forestry. This guideline aims to provide equitable access to trees and green spaces and their benefits by setting the thresholds of having at least 3 well-established trees in view from every home, school, and place of work, no less than a 30% tree canopy in every neighbourhood; and no more than 300 m to the nearest public green space from every residence. Current implementation of this new guideline is discussed, as well as the advantages and disadvantages of using this evidence-based but also clear and simple rules.

## Introduction

With most of the world’s population living in urban areas, there is an increasing focus on making these healthier, more attractive, and more liveable. Although urban areas provide opportunities such as employment, education, recreation, and social interactions, they also face specific challenges. Cities are often hardest hit by climate changes through the urban heat island effect and by extreme weather events such as major heat waves (Devisscher et al. 2019). The European Union (EU) recorded more than a third heat-related mortality in the elderly, with 104,000 out of 296,000 global deaths in 2018, a year when northern Scandinavia experienced temperatures over 5 °C warmer than in 1981–2010 (Taylor 2020).

Air pollution is a major threat to urban areas. In 2018, 34% of urban populations of the 27 EU countries (then including the United Kingdom) were exposed to ground-level ozone particles at concentrations above EU health target levels, while 15% were subjected to hazardous PM_10_ particles at levels above the EU daily limit (EEA 2020). With 84% of the population in Europe exposed to PM_2.5_ levels above the maximum suggested by the World Health Organization, up to 125,000 lives could be saved annually if PM_2.5_ concentrations were reduced to safe levels (IS Global 2021).

Public health challenges faced by urban populations can also be linked to risk factors and lifestyle diseases such as stress, cardiovascular diseases, and obesity (WHO 2016, 2017). The COVID-19 pandemic has created an immediate and often devastating public health challenge to cities, with urban areas representing an estimated 90% of all reported cases of the virus (UNSDG 2020).

Urban green spaces and urban vegetation such as street trees in general are widely recognised as nature-based solutions to help address some of these challenges (WHO 2017; UNECE 2022). The numerous contributions of urban green spaces to public health, climate adaptation, pollution reduction, biodiversity conservation, and water regulation are well-documented (Dobbs et al. 2017). During the COVID-19 pandemic, urban green spaces which had not been closed, provided much needed ‘escapes and areas for mental restoration for urban residents. Global research has recorded major increases in the appreciation for and recreational use of urban green spaces (e.g., Weinbrenner et al. 2021).

Unfortunately, despite their recognised contributions to healthier and more resilient cities, urban green spaces and urban vegetation are often under threat. Densification and sprawl often result in the loss or fragmentation of urban nature (Haaland and Bosch 2015). In the United States, the estimated annual urban tree loss is approximately four million, or about 1.3% of the total urban stock (The Nature Conservancy 2017). Urban trees and other vegetation are also under threat from the impacts of climate change (including drought, extreme weather events, and increased wildfires), pests and diseases, and intensive recreational use (Tubby and Webber 2010; Endreny 2018).

A major problem is that not all urbanites have equal access to urban green spaces and the benefits they provide, as demonstrated by various studies (e.g., Watkins and Gerrish 2018; Shiraishi 2022). Marginalised populations often live in neighbourhoods with lower urban tree canopies. A recent study by Zhou et al. (2021) showed that socially vulnerable urban populations typically live in hotter city areas with fewer trees, and enhancing the urban tree canopy will have a significant cooling effect. This lack of ‘tree equity’ led the American Forests conservation organization to develop its Tree Equity Score for all US cities, showing major discrepancies in the provision of urban tree canopy (American Forests 2021).

Fair and equitable access to urban green space has been widely adopted as a policy objective from the local to the global level. The United Nations Sustainable Development Goal 11 for example, includes target 11.7 that states: “By 2030, provide universal access to safe, inclusive and accessible, green and public spaces, in particular for women and children, older persons and persons with disabilities” (United Nations 2022). Access to urban nature is increasingly seen as an essential service that cities should provide to their residents and has been conceptualised as a basic human right (Miles 2022).

In the light of the above, to ensure and optimize equitable access to urban green spaces and their benefits, it is important to develop evidence-based guidelines for policymakers and urban planners. What does research indicate, for example, about ways to optimize green space access and benefits, bringing urban nature into people’s neighbourhoods? How are trees, as key providers of ecosystem services, integrated in this? What is the importance of having a public park or other green space within easy access of the home? This article provides some answers to these questions. It reviews recent evidence of urban tree and green space benefits in relation to visibility, availability, proximity, and accessibility. Next, ways in which this knowledge has been captured in guidelines and norms are discussed. Finally, a new, comprehensive ‘rule’ or guideline for the greening of cities will be introduced.

## Towards a framework for optimizing green space benefits

### How nature impacts human health and well-being

Based on an extensive review of the literature, Marselle et al. (2021) provides a conceptual framework for how biodiversity is linked to human health. They distinguish between the mental, physical, and social dimensions of health and well-being and includes four pathway domains for linking biodiversity to health: reducing harm, restoring capacities, building capacities, and causing harm. While the latter addresses possible negative effects of interacting with nature (e.g., allergies and vector-borne diseases; volatile organic compounds that can contribute to ozone formation, sometimes referred to as ecosystem disservices), the other three pathways highlight the benefits of interacting with biodiversity in different forms, such as moderating temperature and reducing air pollution (reducing harm pathways), promoting mental wellbeing, and encouraging physical activities (building capacities). Hartig et al. (2014) offered a model of nature–health relationships that identified pathways through which the natural environment and contact with nature influences human health. A distinction is made between the natural environment and contact with nature, acknowledging the various encounters people have with the natural environment and how they conceive of and experience nature. The pathways Hartig et al. (2014) distinguish are air quality, physical activity, social contacts, and stress reduction. The model by Bratman et al. (2019) focuses specifically on the effects of natural features on mental health, identifying a succession of four steps: (1) ‘natural features’, referring to aspects of the environment (e.g., type, size, quality) that can influence mental health; (2) ‘exposure’ which relates to the amount of contact with nature; (3) ‘experience’ which focuses on the experiential aspects of nature exposure; and (4) ‘effects’ which relates to the mental health impacts from nature experience.

The exposure to and experience of nature in urban areas varies depending on where one lives (in a green spaces neighbourhood or not), the opportunities for recreation in parks and other green spaces, and our actual uses of and exposure to green spaces (e.g., continuously or incidentally). The ‘reducing harm’ pathway mentioned by Marselle et al. (2021) depends on the tree canopy in our neighbourhoods and streets, as research has shown the cooling and air pollution reduction role of trees and other vegetation.

There are three types of nature exposure: (1) opportunities to *see* urban nature, and more specifically trees and green spaces; (2) exposure to green spaces by *living amongst* trees and other vegetation; and (3) opportunities for *accessing and using* parks and other green spaces for recreational purposes.

### Viewing trees and green spaces

In their review of the literature on the health effects of viewing different types of landscapes, Velarde et al. (2007) identified three main kinds of health effects: short-term recovery from stress or mental fatigue, faster physical recovery from illness, and long-term overall improvement on health and well-being. In urban areas, sceneries with trees and other green spaces are more beneficial than those without. Research has demonstrated the importance of nearby and visible green spaces for mental health and well-being (WHO 2016; Rugel 2019). During the COVID-19 pandemic, many people have been confined to their homes or neighbourhoods, placing even greater importance on trees and other green spaces in gardens and along streets. Seeing green spaces from our windows helps to keep in touch with nature and its rhythms. It provides important breaks from our work and can inspire us and make us more creative. Views of nature have been found to enhance performance and job satisfaction among office workers (Lottrup et al. 2015). Barron et al. (2019) also described the benefits of greening neighbourhood blocks and possible alternative scenarios for doing so. Having trees and green spaces around homes also make viewing urban nature easy and effortless.

### Living amongst trees and green spaces

Living and growing up in greener neighbourhoods has been associated with different health benefits. Jarvis et al. (2022) studied residential exposure to green spaces on early childhood development using a buffer 250-m from postal code centroids in Vancouver, Canada. They found positive associations of green spaces on early childhood development scores for both total vegetation and for tree cover. Tree canopy has been correlated with a range of public health effects, with living in greener environments associated with better mental health and lower all-cause mortality (Van den Berg et al. 2015). Astell-Burt and Feng (2019a, b, 2020) found that higher canopy cover improved sleep patterns and mental health as well as overall health. A canopy cover of at least 30% for all these aspects resulted in higher health benefits. Having shadier neighbourhoods creates meeting places and enhance social interactions (Holtan et al. 2014). Although the evidence on air pollution reduction by urban trees is ambiguous, there are indicators that well-placed trees can reduce local pollution (e.g., Hewitt et al. 2019). Trees also have important impacts on neighbourhoods by their cooling effects, an important benefit in times of climate change and increasing heat waves. Janowiak et al. (2021), in a report on U.S. government policy on urban climate action, found that urban trees have a crucial role in mitigating the impacts of climate change. Ziter et al. (2019) found that local tree canopy should be at least 40% before substantial cooling effects are noted. Rahman et al. (2019) demonstrated the cooling effects of different tree species through shading (the most important cooling effect) and evapotranspiration.

### Using green spaces for recreation

The proximity of green spaces and trees has been associated with various positive health impacts (Astell-Burt and Feng 2019a, b, 2020; Jarvis et al. 2022). Ngom et al. (2016) found that the proximity to green spaces is important for preventing cardiovascular morbidity and diabetes. Numerous studies highlight the importance of proximity and easy access to high-quality green spaces to promote more regular use of these areas. Recreational activities take many forms, from a walk in the park and walking the dog to active sports and games, and from observing nature and other people to engage in social interactions. Three to 500 m, a safe 5-min walk or 10-min stroll is often mentioned as a threshold for frequent recreational use (Toftager et al. 2011; WHO 2016). Based on a review of the evidence, the European Regional Office of the World Health Organization (WHO 2017) recommends a maximum distance of 300 m to the nearest green space of at least one hectare in size. This encourages the recreational use of green space with positive impacts for both physical and mental health.

### Implementation of research findings

To what extent has the knowledge of the importance of viewing, living amongst, and using green spaces informed policies, guidelines, and standards for greening programs and for urban planning at large? The aspect of visible green spaces has probably received the least attention, although views of green spaces are a well-known positive factor in real estate markets (Jayasekare et al. 2019). The City of Frederiksberg in Denmark has included a specific target in its tree strategy program that says that it should be possible to see at least one tree from every residence (Frederiksberg Kommune 2018).

Living amongst green spaces has received considerable attention, especially through specific tree canopy targets which have been set by municipalities, and sometimes other organisations, at the level of the city or the municipality. The USA not-for-profit organisation, American Forests, for example, recommended a 40% canopy cover until a few years ago. It then withdrew this recommendation based on new research and in recognition of the diverse situations of American cities and towns, which will make it difficult to reach 40% (Leahy 2017). Several of the most ambitious cities when it comes to greening, have set a target of achieving a 30% canopy over the next decades, including Barcelona (Ajuntament de Barcelona 2017), Bristol (Bristol Green Capital Partnership 2018), Canberra (Australian Capital Territory 2019), Seattle (Seattle Government 2016) and Vancouver (City of Vancouver 2020).

Canopy cover has not been the only way to set standards/norms for the greenness of neighbourhoods. Several European cities have had developed green space norms. The European Commission (2020), in its EU Biodiversity Strategy 2030, calls for expansive tree planting and enhancing tree canopy in urban areas, but without setting clear targets. In The Netherlands, for example, the government has recommended that municipalities achieve at least 75 m^2^ of green space for new residential development (Atlas Leefomgeving 2021). Of interest are initiatives by cities like Malmo, Sweden to use a scoring system to help ensure sufficient greening in both new and existing residential areas and has developed its ‘Green Space Factor’ for this purpose (Kruuse 2011). The presence of different types of green spaces including trees, green roofs, and water elements come with a specific score dependent on their extent and number. Developers are required to meet certain thresholds for natural green spaces. Inspired by this approach, cities like London in the UK have developed their own versions of this scoring system (Greater London Authority 2017).

Promoting the use of green spaces for recreational purposes, more specifically by ensuring that people have easy access to urban parks and other types of green spaces, has resulted in guidelines and standards at different levels. An influential and increasingly applied recommendation is the one by the European Office of the World Health Organization: residents should have access to a public green space of at least one ha within 300 m from their homes (WHO 2017). Cities across the world have adopted this guideline or one similar (e.g., using 500 m instead of 300 m or referring to a walk of 5 to 10 min from every home) (WHO 2016).

Several standards and recommendations have been developed that combine proximity/distance with green space typologies. A neighbourhood green space needs to be within easy reach, while a larger natural area or forest can be further away. For Flanders, Belgium, Van Herzele and Wiedemann (2003) refer to a government report that provides accessibility standards for different types of green spaces. ‘Living green’ should be available within 150 m of people’s homes, neighbourhood green spaces of at least 1 ha within 400 m, and city forests of 200 ha or more within 5 km. The United Kingdom is one of the countries that has carried out considerable work on proximity standards. Natural England, a non-departmental public body, developed the guidelines “Accessible Natural Green Space Standards in Towns and Cities” (Natural England 2003). This provides maximum distance guidelines for different types of green spaces. For example, everyone in England should have access to a ‘natural green space’ of at 2 ha within 300 m (a walk of minutes) of their residence. Moreover, at least one natural area of 20 ha or more should be available within 2 km from people’s residence and an area of at least 100 ha within a 5-km radius. Specifically for forests, and in recognition of the many important benefits these provide to people, the UK’s Woodland Trust developed its Woodland Access Standard that stipulates that no one should live more than 500 m from a publicly accessible forest/woodland of at least a 2-ha size. A woodland area of at least 20 ha should be available within a radius of 4 km from people’s homes.

Recognition of the various roles that different types of green spaces play also relates to the topic of quality. It is not just about quantity and sizes, but also about the quality of the green space in terms of offering opportunities for multiple uses and experiences, harbouring (bio)diversity, being well-maintained, and meeting the needs of residents of different ages and backgrounds. Programs such as the Green Flag Award (Ellicott 2016) and the Nordic Green Space Award (2022) (Lindholst et al. 2016) provide a detailed standard with a set of criteria to assess the quality of local green spaces. Aspects considered by these programs include quality of maintenance, accessibility and facilities, cleanliness, and the range of experiences offered.

As noted previously, an important consideration is the fair and equitable distribution of trees, green spaces, and the benefits these provide. This aspect of green equity or environmental justice is often included in greening policies and programs in recognition of the often-uneven access to green spaces within urban areas (e.g., EEA 2021). The previous mentioned Tree Equity Score by American Forests (2021) provides a good tool for assessing inequities in urban tree canopy cover.

### Developing better guidance

Few efforts have been undertaken to develop more comprehensive guidelines that combine the importance of visible, living, accessible, and usable green spaces, and that also recognize the key role of trees and canopy cover. One exception is the Singapore Index on Cities’ Biodiversity (Chan et al. 2014). This Index functions as a standard and scoring system for nature in cities. It consists of 23 different indicators on, for example, the proportion of natural areas in a city (indicator 1), connectivity measures of ecological networks to counter fragmentation (indicator 2), native biodiversity in built up areas (e.g., bird species) (indicator 3), and area of parks with natural areas and protected or secured natural areas per 1000 people (indicator 13). For the latter indicator, maximum points are awarded when more than 0.9 ha is provided per 1000 people. Singapore’s comprehensive Index has been implemented in cities across the world, and sometimes cities have modified it into their own version. The Singapore Index has a strong focus on biodiversity and places less emphasis on, for example, climate adaptation and public health benefits, and the connection of these with urban trees. It also does not explicitly focus on the importance of viewing trees and green spaces, living amongst these, or having them accessible for various recreational activities.

### Towards comprehensive guidelines: the 3–30–300 rule

#### The 3–30–300 rule guidelines

Many working in urban forestry, the planning and management of trees and associated vegetation in urban areas, are familiar with Santamour’s 10–20–30 guideline for urban tree diversity (Santamour 1990). He stressed the importance of a diverse urban forest to build resistance to pests and diseases. As a rule of thumb, he argued, a city’s urban forest composition should not have more than 10% of the same tree species, no more than 20% of a single tree genus and should not exceed 30% of the same family. Although this simple rule was meant to provide overall guidance and has been debated in the literature (e.g., Kendal et al. 2014), it became widely adopted in many cities in North America and elsewhere.

It is likely that the simplicity of Santamour’s guideline and its ‘stickiness’ (Heath and Heath 2007) has led to its wide adoption, even when the evidence supporting it was limited. Where decision makers, planners, developers, and residents may get ‘lost’ in the complexity of nuanced, well-elaborated indices, standards and guidelines, easy-to-remember rules can be very powerful, especially when supported by sound evidence. The challenge will be to balance communicative power and simplicity with the complexity and nuancing required to adapt to different urban contexts.

**Fig. 1:**
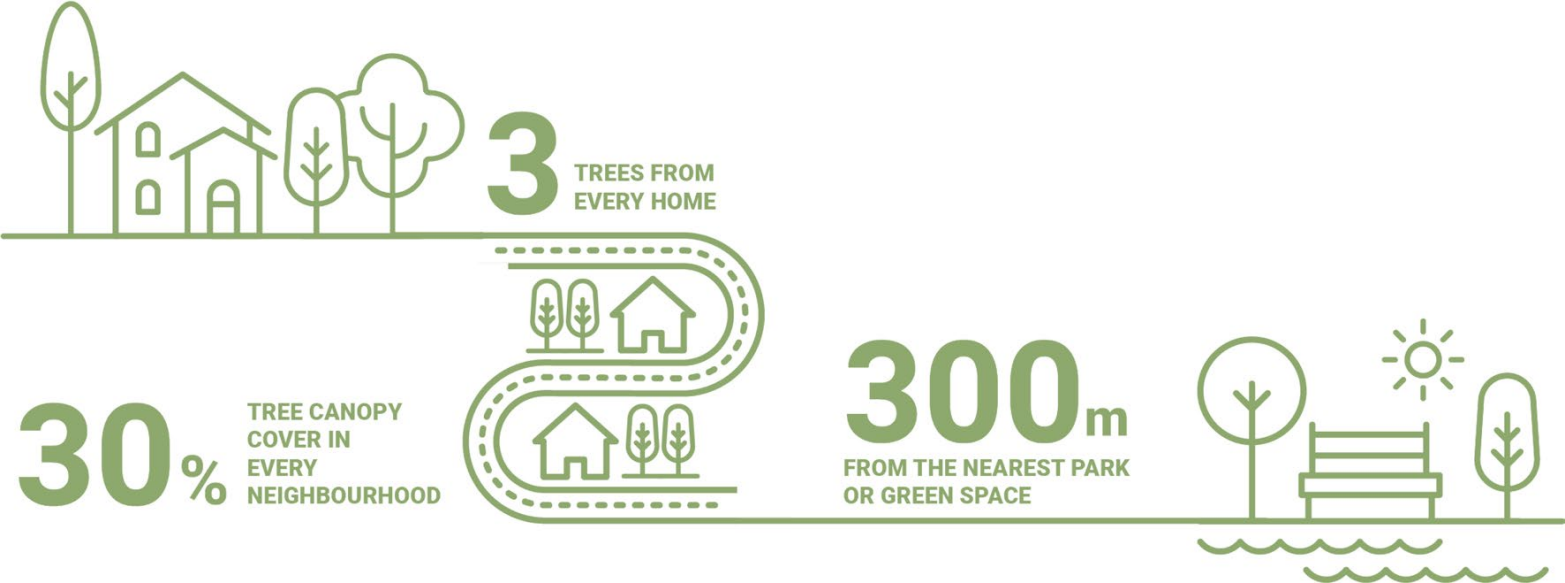
Visualization of the 3–30–300 rule for urban forestry. Source: UNECE (2022), reproduced with permission

Early in 2021, this author proposed a new, evidence-based guideline for developing greener, more resilient, and healthier cities, towns, and neighbourhoods, titled the 3–30–300 rule for urban forestry. The rule builds on the importance of being able to see trees and other green spaces from one’s home (or place of work or learning), living amongst trees and green spaces, and having easy access to nearby public green spaces for recreation. The rule is to a large extent based on the current evidence base, as presented earlier in this paper. It builds on some of the latest evidence that links the visibility, presence, accessibility, and proximity of trees and green spaces to climate adaptation and public health benefits. The rule states that every resident needs to have access to the following (see Fig. 1):

#### The 3 trees from every home, school, and place of work

Every resident in a city, town, or even village should be able to see at least three trees from their home, school, or place of work. These trees should ideally be well-established. Chi et al. (2022) found that fewer large-sized trees impacted resident mental health more positively than a larger number of smaller ones. The three trees can be seen as a ‘proxy’ indicator for visible green space, as trees are widely appreciated by people across cultures (Townsend and Barton 2018) and have considerable presence. Trees play an important role in the visibility aspect of vegetation, as discussed earlier. They also harbour wildlife such as birds and show the rhythms of nature, such as (where relevant) the changing of the seasons. The specific number ‘3’ is not supported by scientific evidence but was chosen to connect with the numbers 30 and 300 from a communication and ‘stickiness’ perspective.

#### The 30% tree canopy cover in every neighbourhood

Based on current research, as previously discussed, at the neighbourhood level, a 30% canopy cover should be a minimum, and cities should strive for even higher canopy percentage when possible. Note that the 30% is not at the city level, as this can result, for example, in tree inequity. Studies have shown the importance of proximity and tree canopy in providing cooling and health benefits, primarily at the local level. Cities like Barcelona, Spain and Vancouver, Canada have canopy cover of around 20% that is heavily dependent on one or more large natural green spaces, but is much lower in the built-up areas of the cities. Thus every neighbourhood needs to be targeted, as well as all new housing developments where there are opportunities to integrate trees from the beginning. Trees provide a wide range of benefits, but in some situations it can be difficult to reach 30% cover with just trees as in existing, dense built-up areas. Where it is difficult for trees to grow and thrive, for example, in arid climates, the green target should be 30% vegetation—but always with a strong tree component.

#### The 300 m from the nearest park or green space

In line with research and with WHO recommendations, every citizen should have a large public green space within 300 m, approximately a 5-min walk or so, from their home. WHO suggests a public green space of at least 1-ha, but this may sometimes be difficult to achieve. The size of green space is important, as larger parks and other green spaces have been associated with more recreational opportunities and higher preferences (Cohen et al. 2010), and with higher levels of biodiversity (Nielsen et al. 2014). It is important to realise that public green spaces come in many different forms and shapes. In Mediterranean cities, for example, treed ‘ramblas’ or walking avenues function as de facto green spaces, even when they are traffic corridors. But it is important that green spaces are of a high quality, allowing for a range of recreational activities, including functioning as social meeting places, and offering opportunities for children’s play. There should also be sufficient trees and vegetation for providing shade.

Since its launch in early 2021, there has been some interest in the 3–30–300 rule from cities and organisations in different countries. Several have formally or informally adopted the rule as part of their urban forestry programs (see for example, Atkins-Baker 2021; UNECE 2022). Using the rule provides evidence-based, easy-to-remember targets that link urban trees and green spaces to climate and health benefits. It also allows for benchmarking (that is, monitoring and comparing with peers nationally and internationally) as well as easy monitoring of progress. The rule’s simplicity makes it easy to communicate and can generate interest and support among residents, politicians, businesses, and other key stakeholders. Applying the 3–30–300 rule can help improve and expand the local urban forests in many cities as part of wider programs and policies, and promote health, wellbeing, and resilience.

Canopy cover and distance to the nearest public green space are easy to assess using various geospatial tools and datasets. In their work for WHO, Bosch et al. (2016) used GIS analysis to assess access the nearest public green spaces for three European cities, showing that the majority of residents in Kaunas, Malmo, and Utrecht live within 300 m of a green space. A study in Germany found that 93% of households have access to green spaces within 500 m and 74% within a 300 m buffer (Wüstemann and Kalisch 2016). The view of trees from homes, places of work, and learning centres is more difficult but here estimates also could be made combining geospatial tools and citizen science, the latter through surveys of a representative sample of people per neighbourhood, asking respondents to report the number of trees they can see. A citizen science approach has the additional benefit of promoting citizen engagement. Various initiatives are ongoing to develop a more comprehensive approach to assessing and monitoring the 3–30–300 rule in North America, Europe, and elsewhere, but none of these have yet been published. The work of Cobra Groeninzicht (2022) in The Netherlands has, for example, used buffers areas around buildings. For the 3 trees component, a buffer of 30 m from the centre of the building may be set, and also use a minimum canopy area (e.g., 25 m^2^) to determine whether trees are ‘in’ or ‘out’. This work has been based on the report by Kluck et al. (2020) on the cooling effects of large versus small canopy trees.

## Discussion and perspective

Guidelines such as the 3–30–300 rule for urban forestry introduced in this paper can be useful for the greening of cities, providing evidence-based direction and offering clear targets as part of policies, programs, and master plans. The development of resilient, healthy, and green urban areas is a complex challenge faced by modern society, and decision makers can benefit from this type of guidance.

Obviously, guidelines are voluntary recommendations and even rules, which are typically compulsory and sometimes even have legal standing, always must be used carefully and with sensitivity to the local context. In the case of the 3–30–300 rule, although the benefits of trees are well-known and their contribution to, for example, cooling effects and health are specific, there may be situations where they may not the right solution. This can be where they may not the appropriate vegetation, where there may be community or cultural reasons for not planting trees, or where neighbourhoods are dense and/or have historical features that make trees inappropriate. A desert city like Riyadh, Saudi Arabia, with a small tree canopy cover cannot be compared with cities like Vancouver that are in a climate where trees grow well. In some cases, cities that have maintained a large canopy over time or that have spread into surrounding forests (‘city in the forest’) while others have lost most of their trees and woodland over time have had to grow their urban forest basically from scratch (‘forest in the city’). There will be important differences in contexts and starting situations but given the essential ecosystem services and benefits trees provide, they should always be part of the discussion, as neighbourhoods are transformed and as new developments need to be critically evaluated for their green components and potential trade-offs between density and greenness. Trees (and shrubs) should be the principal vegetation but sometimes other types of vegetation (such as shrubs, ground cover vegetation) may be more appropriate. The challenge becomes finding combinations of vegetation and green space types that optimise health, climate, and other benefits under specific circumstances. This requires the expertise of urban foresters, arboriculturists, and others to optimise local greening resources.

Guidelines such as the 3–30–300 rule are often the start of a wider discussion on the importance of trees, but also the need to manage and reduce disservices, for example those associated with pollen dispersal and volatile organic compound emissions (Yan et al. 2021). Tree species selection becomes very important to optimise the local use of trees that provide many ecosystem services and limited disservices. Species selection must be related to considerations such as availability, suitability to urban sites, and management needs. This also relates to the need for developing more diverse urban forests of tree and shrub species. Diverse urban forests are more resilient to the impacts of climate change, and pests and diseases, while they also offer variety in terms of experience and perception (Morgenroth et al. 2016).

Guidelines also help with communication and coordination across sectors and disciplines. The 3–30–300 rule has received considerable attention from outside ‘green’ professions, such as planners, engineers, and politicians. The rule has frequently been cited, especially on social media, as ‘an urban planning rule’ rather than an urban forestry or urban greening rule (Quite Interesting 2021). The rule is easily understood by residents and can be used to foster community involvement and stewardship, like the Tree Equity Score achieves in the United States (American Forests 2021). At the very least, the rule will highlight the importance of trees and green spaces in our cities and towns and may initiate debate on the greenness and liveability of the places where most of us live.

Another benefit of guidelines like the 3–30–300 rule is that they are measurable and thus can track progress over time, like what has already been done with some of its components (e.g., canopy cover and distance to the nearest green space). Policy documents such as the EU Biodiversity Strategy 2030 (European Commission 2020) call for all municipalities to developing greening plans, and guidelines such as the 3–30–300 rule could be integrated in these in its current or a locally adapted form. For cities where the 3–30–300 rule is still not feasible, progress can be measured against it, celebrating achieving steps towards ultimately meeting their targets. Where one of the components of the rule is difficult or perhaps even unrealistic, the other components may still be part of policy and program targets. Assessment of the current state of a city’s urban forest according to the 3–30–300 rule, parallel to other possible targets and indicators, can also help with benchmarking with other cities nationally and internationally. Recent years have seen the emergence of various international green city benchmarking schemes including, for example, the European Green City Award (Gulsrud et al. 2017) and the Tree City of the World program (FAO and Arbor Day Foundation 2021). Some of the tools already exist for assessing and monitoring a city’s urban forest program according to the 3–30–300 rule, but further development and testing of methods will be needed.

As the 3–30–300 rule is being adopted and implemented, it will be important to closely monitor its impact and the role it plays in greening cities and towns. Peer networks between cities and organizations can assist with mutual learning, as can the identification and communication of case studies and pilot projects—both successful and less so. The rule is a guideline that needs to be carefully implemented, making adjustments to local realities when needed and being aware of wider discussions on urban tree benefits and potential ecosystem, disservices and the need for diverse and resilient urban forestry programs. Studying how the 3–30–300 rule works in different climates is an important component of this.
